# Integration of Human Papillomavirus Genomes in Head and Neck Cancer: Is It Time to Consider a Paradigm Shift?

**DOI:** 10.3390/v9080208

**Published:** 2017-08-03

**Authors:** Iain M. Morgan, Laurence J. DiNardo, Brad Windle

**Affiliations:** 1 Philips Institute for Oral Health Research, Virginia Commonwealth University (VCU) School of Dentistry, Department of Oral and Craniofacial Molecular Biology, Richmond, VA 23298, USA; bwindle@vcu.edu; 2 VCU Massey Cancer Center, Richmond, VA 23298, USA; laurence.dinardo@vcuhealth.org; 3 VCU Department of Otolaryngology, Richmond, VA 23298, USA

**Keywords:** head and neck cancer, human papillomavirus, human papillomavirus 16, HPV16, The Cancer Genome Atlas, oral keratinocytes, integration, episomal, mixed

## Abstract

Human papillomaviruses (HPV) are detected in 70–80% of oropharyngeal cancers in the developed world, the incidence of which has reached epidemic proportions. The current paradigm regarding the status of the viral genome in these cancers is that there are three situations: one where the viral genome remains episomal, one where the viral genome integrates into the host genome and a third where there is a mixture of both integrated and episomal HPV genomes. Our recent work suggests that this third category has been mischaracterized as having integrated HPV genomes; evidence indicates that this category consists of virus–human hybrid episomes. Most of these hybrid episomes are consistent with being maintained by replication from HPV origin. We discuss our evidence to support this new paradigm, how such genomes can arise, and more importantly the implications for the clinical management of HPV positive head and neck cancers following accurate determination of the viral genome status.

## 1. Introduction

Recently we published an analysis of head and neck cancer data from The Cancer Genome Atlas (TCGA) providing evidence that the three types of genomic status for human papillomavirus (HPV) in head and neck cancer (HNC) are episomal, integrated and virus–human hybrid episomes replicating autonomously; there was very little evidence for the presence of so-called mixed tumors that have both episomal and integrated viral genomes [1]. In this review, we will describe HPV replication and the mechanisms that could promote viral genome breakage and therefore association with host DNA. We will then discuss the current status of understanding of HPV integration in head and neck cancer and explain why our results are not in conflict with those of others. The implications of our model for management of HPV-positive HNC will be described, particularly for individuals who are receiving de-escalation therapy in clinical trials. Finally, we will propose a set of diagnostic assays for predicting the genomic status of HPV, and the clinical importance of this status.

## 2. Human Papillomavirus Replication

The HPV life cycle is inextricably linked to the differentiation of the host epithelium [2,3]. Infection begins with targeting of basal epithelial cells, thought to be stem cells, followed by entry of the viral genome into the cell nucleus that requires mitosis of the infected cell [4,5]. Cellular factors then interact with the long control region (LCR) of papillomaviruses to activate transcription from the viral genome [6] and this results in expression of the viral genes and proteins. In high-risk HPV (HR-HPV, that causes cancer), E6 and E7 are viral oncoproteins that target the tumor suppressor proteins p53 and pRb [7] and this promotes replication of the infected cell, which then migrates through the differentiating epithelium. The initial phase of replication in the infected cells is called establishment, where the viral genome copy number increases to 20–50 copies per cell. Therefore, during this process the viral replication process is not under a strict once and once only per cell cycle control. By definition, the HPV has to overcome this limitation. The second replication phase of the viral life cycle is called the maintenance phase where, during the differentiation and proliferation of the infected cell, the viral genome copy number is controlled at 20–50 copies. Finally, in the differentiated epithelium when cell proliferation has been arrested the viral genome amplifies to around 1000 copies per cell before being encapsulated by L1 and L2 prior to viral particle egress from the cell. Given the number of genomes generated during the establishment and amplification replication phases, viral replication is very different from that of the host. This difference includes evasion of the tight control over host cell replication timing and quality.

There are two viral proteins that coordinate viral replication for all papillomaviruses in association with host proteins; E1 and E2. The E2 protein forms homodimers and binds to 12-bp palindromic DNA sequences surrounding the viral origin of replication in the LCR and recruits the viral helicase E1 to the origin of replication [8,9]. E1 interacts with host polymerases and other factors involved in DNA replication including single-stranded binding proteins [10,11,12,13,14], while E2 also interacts with cellular proteins to promote viral replication including TopBP1 [15,16,17,18], Brd4 [17,19] and ChlR1 [20].

## 3. Human Papillomavirus Replication and the DNA Damage Response: Primed for Integration

High-risk HPV activate a DNA damage response (DDR) that promotes the viral life cycle and several DDR proteins are involved directly with HPV replication [15,16,21,22,23,24,25,26,27]. The E7 protein elevates the levels of proteins involved in the DDR [28] while the E1 helicase activates a DDR when expressed in cells [29,30,31]. It also seems likely there is a combination of activation of the DDR by E1 combined with E7-mediated elevation of factors involved in this process. Therefore, HR-HPV cells are unusual in that they replicate and go through a cell cycle while a DDR is turned on in the cells; ordinarily the DDR that is activated in response to genotoxic stress arrests the cell cycle to promote repair of DNA damage. One proposed benefit to the virus for activation of the DDR is to recruit factors that promote homologous recombination to the viral genome in order to promote the amplification stage of the viral life cycle [32,33]. Significantly, DNA damaging agents do not arrest E1–E2-mediated DNA replication, even if the host cell replication is arrested, perhaps due to E1 not being a substrate for ATR/ATM [29,34]. This is not surprising as the virus activates the DDR to promote its own life cycle; inhibitors of the DDR block the viral life cycle [22] and therefore the virus must be able to replicate in the presence of DNA damage. However, the E1–E2-mediated DNA replication that occurs in the presence of DNA damaging agents is extremely mutagenic [29] even though the replication levels are not affected. The precise reason behind the requirement of an activated DDR to promote the HPV life cycle remains unclear but it does build in a degree of viral genomic instability into the viral life cycle. If the infected cell is genotoxically stressed, then the viral replication will continue and this replication in the presence of DNA-damaging agents could promote double-strand breaks in the viral genome. Such breakage would provide a substrate for integration into the host genome via non-homologous end joining mechanisms. Indeed, integration is observed in HR-HPV positive cancers.

## 4. Human Papillomavirus and Integration in Cervical Cancer

A large majority of cervical cancers are caused by HR-HPV [35]. There are a number of HR-HPV genomes that are causative in cervical cancer; HPV16 is present in around 50% while HPV18 is present in around 20%. The first demonstration that the HPV genome was adjoined with human DNA was made using cervical cancer DNA samples and also cervical cancer cell lines [36,37,38,39,40,41,42,43,44,45,46,47,48,49,50,51,52]. Originally this was primarily confirmed using Southern blotting of DNA digested with enzymes that cut the HPV16 genome in a single position or not at all. In many cases the disruption in cancer samples was in the E1 and E2 genes and this could also be observed in cell lines with HPV16 genomes; lack of E2 is associated with more aggressive tumor and cell line growth [53,54,55,56,57,58,59]. Overexpression of E2 can repress HPV LCRs in transient transfection experiments [60,61,62], therefore it was originally proposed that the loss of E2 is required to increase E6 and E7 expression and subsequent progression of the transformed cell to a more malignant phenotype. E2 has no effect on transcription from episomal HPV16 DNA in W12 cells (a cell line containing HPV16 derived from a cervical lesion) [63], although it can repress transcription from integrated genomes in cervical lines [63,64,65,66,67]. However, given the toxicity of the E1 protein in cells due to binding to and unwinding of host DNA resulting in a DDR [30,31], it is equally possible that the expression of E1 must be disrupted in order for the integrated cells to survive. E1 and E2 expression from integrated DNA would initiate DNA replication from the integrated viral LCR and this would create replication stress within the host genome throughout S phase and beyond as E1 and E2 replication is not under the control of host DNA rules with regards to a once and once only per cell cycle replication firing. However, there is a mechanism that allows for the presence of the E2 and E1 genes in tandemly integrated HPV genomes and that is methylation of the viral DNA [68,69,70,71,72,73,74]. In derivatives of W12 cells with integrated DNA there are two types that occur; Type 1 has only one copy of the viral genome and that is non-methylated while Type 2 has tandem integrants that are methylated to silence the viral genome, only one copy produces E6/E7 transcripts and there is no E1/E2 expressed. There are two cell lines that model this, SiHa has one copy of an integrated genome that is hypomethylated, while CaSki has multiple genomes integrated that are hypermethylated [75]. When methylation is reversed in CaSki cells using 5-aza-2′-deoxycytidine the cells die, perhaps due to overexpression of other viral genes such as E1 and E2.

## 5. Human Papillomavirus and Head and Neck Cancer

Head and neck cancer is the sixth most common worldwide, with around 600,000 cases diagnosed annually. Cancer incidence in the developed world is decreasing in general, due to smoking cessation; the one exception to this is the increasing cases of head and neck cancer. This increase is due to an increase in the incidence of HPV-positive head and neck cancer in the oropharyngeal region (HPV + OPC, oropharyngeal cancer) over the past several decades; HPV16 is causative in 80–90% of these tumors [76,77,78,79,80,81]. The incidence of HPV + OPC continues to increase [81]. The 5-year overall survival of patients with HPV + OPC is increased over two-fold when compared with HPV-negative head and neck cancers, and this is attributed to a good response to chemo–radiation therapy (CRT) [82,83,84]; the precise reason for the improved response of HPV + OPC to CRT is not clear. There are no diagnostics (such as Pap smears for cervical cancer) available for assisting with the management of HPV + OPC and no therapeutics available that directly target the viral life cycle.

## 6. Human Papillomavirus and Integration in Head and Neck Cancer

As described above, integration of the HPV genome into the host is a common feature of cervical cancer, and integration is an indicator of a poorer clinical outcome. The situation with HPV integration in head and neck cancer with relation to clinical outcome is much less clear. Early studies on HPV and integration in head and neck cancer employed fluorescence in situ hybridization (FISH) applied to interphase nuclei to visualize the viral genomes and concluded that the appearance of defined “dots” in the nuclei were indicative of viral genome integration [85,86]. This FISH approach was based around a report that suggested dispersed FISH staining indicates episomal genomes while punctate FISH staining suggests integrated viral genomes [87]. Other studies assumed the loss of the E2 gene during integration, as happens in cervical cancer, and measured the ratio between the E2 and E6 genes; an E2/E6 ratio of less than 1 is presumed to contain integrated DNA [88,89]. Using these approaches, investigators concluded that the majority of HPV-positive head and neck cancers had integrated viral genomes, similarly to cervical cancer. An additional approach for investigating viral genome integration is to carry out Amplification of Papillomavirus Oncogene Transcripts PCR (APOT-PCR) and also Detection of Integrated Papillomavirus Sequences PCR (DIPS-PCR); both of these techniques search for the association of viral with human DNA. These techniques were used on HPV-positive head and neck cancers and combined with analysis for the expression of the E6 and E2 viral genes [90]. This study identified 39% of the tumors with integrated DNA, while the remaining 61% had episomal viral genomes. In addition, even though they could see integration of the viral genome with loss of E2, they could still detect E2 transcripts in some of these tumors which they postulate is due to the presence of episomal viral genomes alongside the integrated DNA. Our own analysis indicated that ca. 66% of HPV-positive head and neck cancer samples had HPV joined to human DNA, however, our results lead us to a different interpretation of what these HPV–human DNA junctions represent.

Full RNA and DNA genomic sequences for HPV-positive head and neck cancers became available following publication of data from The Cancer Genome Atlas Network [91]. This original report from TCGA concentrated on the gene changes and mutations of the tumors and was not focused on the HPV status of the tumors; however, they did conclude that gene expression levels of genes associated with HPV integration were higher than those detected in corresponding non-integrated HPV-positive head and neck cancers. A subsequent publication from TCGA Network did focus on the status of the HPV genome in these tumors [92], carrying out the analysis of 279 tumors, 35 of which had evidence of HR-HPV types 16, 33 or 35. From this analysis they determined that 25 of these cases had evidence for integration of the viral genome into that of the host in regions that were more likely to be associated with genes. However, there was no strong association with any particular host gene although the limited sample size may be an explanation of the failure to find such an association. They also concluded that integration was not associated with clinical outcome, unlike for cervical cancer where integration does statistically result in a worse clinical outcome. Several other studies looking at HPV integration in head and neck cancer also determined that viral genome integration did not result in a worse clinical outcome [93,94].

Recently we published a report detailing our genomic analysis of all of the head and neck cancer samples from The Cancer Genome Atlas [1]. Here we will summarize these findings as it is pertinent to the main thrust of this review, that there is an alternative interpretation of the genomic structure of HPV in head and neck cancer. In this manuscript, we analyzed data from all 520 head and neck cancer samples and determined that 72 were HPV-positive as determined by viral gene expression and of those, 83% had HPV16 which we focused on for this report. After considering the HPV16 status and the availability of RNA-seq and DNA-seq data we analyzed 30 tumors in depth. At the genomic level, there were three types of tumors: (1) those that had deletions in the E1–E2 region of the viral genome suggesting an integration event; (2) those that had a constant number of reads across the entire HPV16 genome suggesting an intact genome and therefore an episomal genome; and (3) those that contained DNA covering the entire viral genome but that had a portion of the genome represented at around half the reads as the rest of the genome. This latter category could be arrived at if there was a mixture of integrated and episomal viral genomes present in the tumors. However, the striking feature of these tumors was the copy number for supposed integrated HPV and the copy number for supposed episomal HPV was ordinarily equivalent (there was as many predicted integrated genomes as there were predicted episomal). This was true for a broad spectrum of copy numbers, from 5 to 130. This struck us as unlikely to represent truly mixed tumors that contained episomal and integrated versions of the viral genome whether in the same cell or separate cells; one might expect the episomal genomes to be in large excess over the integrated numbers, but certainly not conspicuously equal at a statistically significant level. Another striking feature of our analysis of the three types of genomic HPV16 tumors was that in the purely integrated samples there was a low number of viral genomes per cell. This is to be expected for an integrated tumor; the median copy number was 1.7, compared to greater than 14 for episomal HPV samples. The RNA-seq data from the tumors confirmed that Category 1 tumors were truly integrated as there was no viral RNA transcript that went past the proposed integration site. There was a truncation of transcription within the E1 gene in all Category 1 samples, thus no functional E1 was produced and there was no expression of E2, E4, or E5. For the other two categories of tumors, the HPV genome was expressed intact throughout the early region with no deletions or differences in the levels of the particular viral genes between the two categories.

Analysis of the DNA-seq data supported our conclusion that Category 1 tumors were integrated tumors as hybrid viral–human reads that describe the integration point could be detected. Category 2 tumors were confirmed as predominantly episomal as there was no consistent detection of viral–human reads. The conclusion about our Category 1 and 2 tumors is no different from that arrived at by others. The difference in our interpretation is with the Category 3 tumors. Others would have called this a “mixed” tumor that contains both episomal and integrated viral DNA. This concept is difficult to reconcile with the observation that E1 can bind to and amplify DNA when it is integrated into that of the host [30,31,95]. Therefore, if there was a viral genome containing an origin of replication integrated into the host, and E1 and E2 expressed from an episome in the same cell, there would be nothing to prevent the viral replication complex recognizing the origin in the host genome and initiating DNA replication. This would be toxic; during initial infection E1–E2 replication is not controlled by a once and once only rule as is host replication, therefore repeated initiation of replication from host associated DNA would result in genome breakage and activated DNA damage response and repair machinery. In the long term, the presence of both integrated and episomal viral genomes in the same cell seems incompatible for this reason. All samples we assessed as integrated in our study had no expression of intact E1 and E2, consistent with this scenario. It is not clear from cervical cancer studies, where the presence of “mixed” tumors has also been proposed, whether the integrated and episomal genomes exist in the same cell in the tumor or whether they exist in separate cells within the same tumor. This is also a possibility for the head and neck cancer samples. However, the maintenance of the same number of both integrated and episomal genomes in separate cells in most samples is unlikely. Therefore, we considered alternative interpretations of the status of the HPV genome Category 3 tumors.

**Figure 1:**
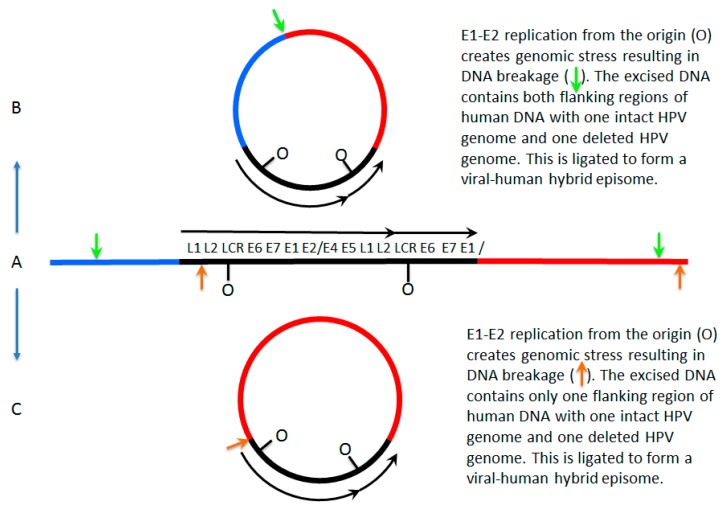
Mechanisms for formation of viral–human hybrid episomes. In (**A**), a human papillomavirus 16 (HPV16) dimer has broken and integrated into the host genome, losing a part of one of the viral genomes as often happens during integration. E1 and E2 can still be expressed from the intact HPV genome and therefore the potential to initiate replication from the viral origins is retained; this initiation is not restricted to once per cell cycle therefore repeated initiation would form an “onion skin” replication bubble. This would create stress on the host genome resulting in double strand breaks promoting excision from the host genome. This viral–human hybrid DNA could then be ligated to form an episome. In (**B**), the breaks occur in the host genome flanking the integrated viral genome (the green arrows) and ligation occurs to form an episome that consists of the viral genome with two flanking regions of human DNA. In (**C**) the breaks in the DNA occur in the viral genome and in flanking human DNA (orange arrows) and ligation occurs to form a viral–human hybrid episome that consists of the viral genome with one flanking region of human DNA. LCR = long control region.

Our conclusion from analyzing TCGA Whole Genome Sequencing (WGS) data for Category 3 tumors is that the virus genome is not integrated into that of the host genome. Rather, the HPV genome replicates as an independent viral–human hybrid episome. This suggests that at some point the original episomal viral genome broke and ends joined to that of the host but subsequently the host DNA associated with the HPV DNA broke and a ligation event occurred resulting in excision of DNA that forms a viral–human hybrid circular episome. Potential mechanisms to explain this are shown in Figure 1. In both cases shown in this figure, E1 and E2 would still be expressed and would initiate replication from the viral origin of replication. This initiation is not controlled in cell cycle manner therefore multiple initiation events could occur forming an “onion skin” replication structure that could be resolved by double-strand DNA breaks. Following breakage of the host DNA the linear viral–human DNA could be ligated to form a circular viral–human hybrid episome. There are multiple pieces of evidence that support the presence of these viral–human hybrid episomes. Firstly, genomic DNA for HPV and joined human DNA maps as a contiguous circular structure, not a linear structure. Secondly, viral-associated human DNA is equivalent in amplification to that of viral DNA, which would be expected if within an episome. Thirdly, there were novel human–human DNA junctions mapped that represent the excision and joining of ends to form the viral–human hybrid episomal DNA elements (the green arrow in Figure 1B). The copy number for these hybrid human–human junctions are the same as of the viral genome structures and for the amplified associated human DNA. Fourthly, the viral–human DNA junctions had the same prevalence as the viral genomes; if there were multimers of the viral genome integrated into the host then this number should be much lower. Overall the evidence overwhelmingly suggests that in samples people have considered “mixed” tumors, the virus in fact is episomal and replicates joined to a segment of human DNA.

The conclusion from our analysis is that the viral genome is maintained episomally either as an intact HPV genome structure or an HPV–human DNA hybrid episome. If the HPV genome co-exists as both an integrated and episomal structure in tumors, it is not common. In Section 7 we discuss why our conclusions are not incompatible with the work of others, but that the differences are in interpretation of the data. TCGA data has been crucial to the development of our model as it provides much more in-depth information than simple diagnostic assays that have been used in the past to characterize the viral genome in head and neck cancers.

## 7. A Model for Integration and Excision of HPV DNA

The proposed initial driving force for integration is DNA breakage in which HPV is linearized in the E1 region and some DNA is removed from the ends to result in deletion. Breakage of the human DNA then leads to recombination of the free ends of the HPV DNA with the free ends of the human DNA, resulting in integration. For a monomeric HPV episome, the integrated HPV DNA will have lost the ability to express an intact E1 and any E2. The integrated HPV DNA will no longer initiate replication from its own origin within the LCR and will be replicated from human origins as part of the human genome. However, if the same breakage and integration events occurred for a dimeric HPV episomal genome, the result could be quite different. An integrated dimer of HPV DNA will retain the ability to express E1 and E2 from the unaltered copy of the HPV genome. This would allow the initiation of replication from the HPV origin while being within the human genome. Since HPV-origin firing can evade the one initiation per cell cycle control used for human origins, the HPV genome could go through multiple rounds of replication in a single cell cycle, which is predicted to form the proposed onion skin structure causing harm to the cell. This problem can be averted or solved by the excision of the HPV DNA to form a new episome. While we propose the excision encompasses the HPV DNA, there are possible options as to what DNA is excised. The HPV episome could be formed from excision of human DNA on both sides of the integrated HPV DNA encompassing all HPV DNA. The excised DNA could encompass human DNA on only one side of the integrated HPV DNA and most of the HPV DNA. Figure 1 summarizes these mechanisms graphically.

## 8. Conflicting Interpretations Rather Than Conflicting Results

The question that arises from our work [1] is why have others not observed this before, or why this interpretation has not been made before in head and neck cancer? One of the answers lies in the fact that there have not been a large number of studies in this area with clinical samples. Sequencing of head and neck cancer lines have been done and revealed a host of genomic changes associated with integrated HPV genomes [96]. However, in head and neck cancer cell lines we suggest that episomal HPV, whether by itself or as a viral–human episome, integrates into the host genome during culture; such an observation is common in cervical cell lines containing episomal HPV16 genomes [58]. Therefore, cell lines may not be an accurate model for studying the physical status of the HPV genome in actual cancers. Some studies have used APOT-PCR [90] and detected viral–human transcripts suggesting integration. However, such transcripts would also be detected in hybrid viral–human episomes, therefore they do not prove definitively that the viral genome is permanently integrated into that of the host. Indeed, in this particular study they observed deletion in the E2 region but the presence of E2 transcripts and concluded the presence of mixed tumors. However, their data is not incompatible with the existence of viral–human hybrid episomes that have lost one of two copies of the E2 gene. A standard approach for the characterization of the HPV genome status in both cervical and head and neck cancer is to measure the ratio of E2 DNA to E6 DNA and declare that if that ratio is less than one then there must be an integration event [88,89]. If the ratio is zero (i.e., there is no E2 DNA) then it would be consistent with an integrated tumor. However, in these types of studies it is common to observe ratios around 0.5 similar to our Category 3 tumors. If the virus replicates as a dimer and loses a copy of the E2 gene, then the ratio of E2 to E6 would be 0.5. Therefore, using an E2 to E6 ratio from PCR is not a good indicator of HPV genome integration. One of the earlier ways that the integration was predicted was to use FISH analysis where it was suggested that punctate staining of the viral genome indicated an integrated tumor while more dispersed staining indicated an episomal genome [87]. However, this is not a reliable mechanism for truly detecting integration. For example, Parvenov and colleagues [92] suggested that there may be viral–human structures in HPV-positive head and neck cancers and to investigate this used a FISH approach for the human DNA and observed punctate staining. It is of course possible that in this clinical sample the viral–human hybrid DNA is indeed integrated, but integration would have to have occurred in multiple points on the human genome as there were multiple FISH signals detected. In differentiating cervical cell lines that retain HPV episomes, the viral genome is amplified in large replication foci [32] and when this happens they do look punctate and would be interpreted as integrated using this classification. They are not. In addition, we also see in oral keratinocytes containing episomal HPV16 genomes the presence of detectable discreet viral foci by FISH even in non-differentiated epithelial cells [97]. These would be interpreted as integrated using this criterion, but they are not. Therefore, the punctate/dispersed staining criterion is not a reliable mechanism for truly predicting whether the viral genome is integrated or episomal.

**Figure 2:**
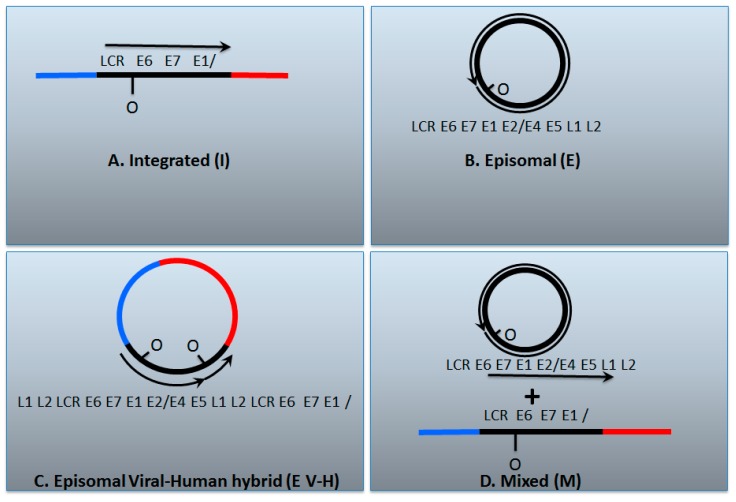
The genomic status of HPV16 in head and neck cancers. (**A**) The HPV genome has broken and become integrated permanently into the host; (**B**) The HPV genome remains as an episome; (**C**) An HPV dimer (or multimer) has integrated and been excised along with human DNA and ligated together to form a viral–human hybrid DNA episome (see Figure 1); (**D**) Previously the viral–human hybrid episome tumors would have been assigned as a mixed tumor that contains both integrated and episomal viral genomes. The box below describes various tests that have been used to characterize the status of the HPV genome in cancers; Y = yes, N = no. It is notable that all of these tests carried out together can differentiate between integrated and episomal tumors, but they do not allow differentiation between whether the viral genome exists as an episomal viral–human hybrid or as a mixed tumor. We propose that these tumors have viral–human hybrid episomes following our analysis of The Cancer Genome Atlas data; so-called mixed tumors, if they exist at all in head and neck cancers, are rare. See the text for details.

We are not claiming that the work of others is wrong in any way. What we are proposing is that, in light of data from The Cancer Genome Atlas, it may be worthwhile re-interpreting older results. Our results are not in conflict with those of others. Figure 2 summarizes these thoughts.

## 9. Why This Matters: De-Escalation Therapy for Human Papillomavirus-Positive Head and Neck Cancer

HPV-positive head and neck cancer patients have a much improved overall survival when compared with HPV-negative patients [82]. For this reason, it has been proposed to treat HPV positive patients with a de-escalated therapy [98] with the aim of reducing the cytotoxic effects of CRT on these patients; there are ongoing clinical trials in this area. However, although HPV-positive head and neck cancers do in general respond better to therapy, a significant percentage do not, varying from 10 to 20% of patients [99]. Therefore, in planned de-escalation therapy it is important to be able to identify those HPV-positive patients who are predicted to not respond well to therapy; at the moment, there is no way to do this. p16 staining in HPV-positive head and neck cancers is the best prognostic marker for predicting clinical outcome; traditional staging parameters based around primary tumor extension, lymph node involvement and distant metastasis historically had no prognostic value for HPV-positive head and neck cancers [100]. More recent attempts at staging HPV-positive oropharyngeal cancers have met with some success in being able to predict worse clinical outcomes, but none of these systems offers a guaranteed path for identifying patients who will definitely fare worse clinically although there are detectable trends [101,102,103,104]. Almost all HPV-positive tumors exhibit p16 overexpression, therefore this marker is not able to identify those HPV-positive patients who will not respond well to therapy. For these reasons, even though there are ongoing de-escalation clinical trials, the recommended standard treatment for HPV-positive head and neck is no different from that used for HPV-negative patients.

One indicator for worse outcome in HPV-positive cervical cancer is integration of the viral genome into that of the host. However, several reports have claimed that this is not the case in HPV-positive head and neck cancer [93,94]. We propose that mischaracterization of integrated tumors is the reason for this and that in fact, those tumors that do have truly integrated viral genomes are those that do worse clinically. Our preliminary analysis of clinical outcomes for TCGA tumors based on our categorization of those that are truly integrated (Category 1) demonstrates that integration does predict a worse outcome (in preparation). Those tumors that we describe as Category 3, where there are viral–human hybrid genomes replicating as an episome, do as well clinically as Category 2 tumors, which have virus-only episomes. Previous studies in this area are confused by the definition of integration being defined by E2 to E6 ratios and/or the conclusion that “mixed” characterized tumors with integrated and episomal genomes co-existing exist. These studies would include Category 3 tumors (virus–human episomes) as having integrated viral genomes (“mixed”) along with the truly integrated Category 1 tumors. It would be interesting for the authors of these reports to go back and reanalyze the data by including their “mixed” tumors as being predominantly episomal, or reanalyzing the samples using different techniques.

## 10. Future Approaches to the Diagnostic Management of Human Papillomavirus-Positive Head and Neck Cancer

What is the best approach for characterizing tumors that have truly integrated viral DNA? We can easily eliminate some tests. Measuring an E2 to E6 DNA ratio as an indicator of integration is invalid for several reasons. Firstly, there are several head and neck tumors where the integration of the viral genome is in the E1 gene and the E2 gene is actually retained intact, therefore a 1:1 ratio would falsely suggest these tumors were episomal. Secondly, in our model we show that the viral genome exists as dimers/multimers in episomal forms and that in some occasions the E2 gene from one of the viral genomes can be deleted. This would give an E2 to E6 ratio of less than 1 suggesting integration but this would not be the case. Thirdly, in our Category 3 tumors where we propose the virus is replicating as a viral–human episome it is a viral dimer/multimer that fuses to the host genome and again E2 can be lost in one of the copies of the viral genome. Therefore, in these tumors the E2 to E6 ratio would be less than 1 but the viral genome is not permanently integrated into that of the host. Another technique that has been used to confirm the presence of integrated and/or mixed tumors is Southern blotting. But again, in our model the results from such analysis are open to misinterpretation. For example, if a viral–human hybrid episome was digested with *Bam*H1 (a single cutter for HPV16) and bands are seen at 8 kbp and additional positions on the gel it is presumed that this represents a mixed tumor. The proposed viral–human hybrid genomes could give the same signal. In addition, Southern blotting of tumor DNA that does not cut the viral genome has been used to suggest integration but again, if the human DNA replicating with the virus in a hybrid episome is cut by the non-cutter enzyme confusing results can occur in addition to the size of the uncut virus–human hybrid also confusing the results. Two other assays that are used, APOT-PCR and DIPS-PCR, are used to monitor the presence of viral-human RNA and DNA products, respectively, and have been used to demonstrate integration. However, these techniques would give positive signals in Category 3 tumors where the viral genome is replicating as an episome as a viral–human hybrid. FISH has also been used to indicate the presence of integrated HPV DNA but this technique can also pick up episomal viral DNA and does not precisely define integration. Therefore, none of these techniques are appropriate for categorizing the genomic status of HPV in head and neck cancer.

From our preliminary analysis of outcome data from TCGA HPV-positive head and neck cancers the data demonstrates that our Category 1 patients (those that we predict only have integrated DNA) do worse clinically than those with tumors containing the virus as an episome, whether replicating with human DNA as a hybrid or not. This makes our challenge easier in many ways as, for clinical outcome concerns, a simple identification of HPV-positive tumors with truly integrated DNA is required. A simple assay for identifying these patients who are truly integrated is the absence of E2 through to E5 RNA. Indeed, a recent report looking at just this facet of HPV positive head and neck cancers demonstrated that, using the lack of E2 RNA as an indicator of integration, patients who lacked E2 expression had poorer clinical outcomes [105]. Therefore, a simple RNA in situ hybridization probe of formalin-fixed, paraffin-embedded clinical samples would allow stratification of HPV-positive tumors into integrated and non-integrated types; the presence of E6 RNA and the absence of E2/E5 RNA would predict integration. Such a simple characterization could be done with existing tumor samples allowing for outcome data analysis.

Therefore, we propose that as part of de-escalation clinical trials, the status of HPV-positive head and neck tumors with regards to E2/E5 expression should be considered. Patients who lack E2/E5 expression should be followed very carefully in such trials.

## 11. Conclusions

Looking at the head and neck cancer data from TCGA resulted in our model of the three different types of HPV genome status. Considering all possible explanations of what is present in the data, we do think it reasonable that “mixed” tumors are a category where the viral and human sequences replicate together as an episome. This matters as including this category as having integrated viral genomes has muddied the interpretation of outcome for HPV-positive patients. During de-escalation trials, it is important to identify patients who are at increased risk and we predict that these patients will be those who have truly integrated tumors. Much work remains to be done. We are currently working on establishing cell lines from head and neck cancers where we will look at the status of the viral genome at very early passage to ensure that the viral genome does not become integrated following medium-term cell culture. We are in the process of using E6/E2/E5 RNA in situ hybridization with tumor samples to determine if those that lack E2/E5 expression do have worse clinical outcomes. We conclude by asking the HPV and clinical communities to have an open mind with respect to our proposed model and consider it for testing.

## Figures and Tables

**Table:** 

	**I**	**E**	**E V-H**	**M**
E2/E6 DNA ratio is less than 1	**Y**	**N**	**Y**	**Y**
Viral–human DNA hybrids	**Y**	**N**	**Y**	**Y**
Viral–human RNA hybrids	**Y**	**N**	**Y**	**Y**
Full length E2 RNA expression	**N**	**Y**	**Y**	**Y**
E5 RNA expression	**N**	**Y**	**Y**	**Y**
8-kbp band on Southern blot following digestion with single cutter	**N**	**Y**	**Y**	**Y**
